# Safety of a 90-min duration of intravenous infusion of obinutuzumab in patients with B-cell non-Hodgkin's lymphoma in a tertiary hospital in China: a prospective, open-label, exploratory clinical trial

**DOI:** 10.1590/1414-431X2023e13284

**Published:** 2024-02-09

**Authors:** Shuangshuang Xing, Yiqin Pu, Xiaoqian Zhao, Yan Hu, Feiyan Zhang, Zejuan Gu, Wei Xu, Lei Fan, Yi Miao, Jianyong Li

**Affiliations:** 1 Department of Hematology, The First Affiliated Hospital of Nanjing Medical University, Jiangsu Province Hospital, Nanjing, China; 2 Department of Nursing, The First Affiliated Hospital of Nanjing Medical University, Jiangsu Province Hospital, Nanjing, China

**Keywords:** Obinutuzumab, Rapid infusion, Intravenous infusion reaction, Care

## Abstract

This study aimed to analyze the safety and applicability of a 90-min duration of infusion (SDI) of obinutuzumab in patients with B-cell non-Hodgkin's lymphoma (NHL) in a tertiary hospital in China. This exploratory clinical trial was performed at Jiangsu Province Hospital. All patients were treated with the standard infusion regimen for the first infusion. If no grade ≥3 infusion-related reactions (IRRs) occurred, the subsequent infusions were given as SDI. The primary endpoint was the incidence of IRR during the standard infusion (3-4 h) and 90-min SDI regimens. This study enrolled 208 patients and all completed cycle 1. Forty-one patients (19.71%) had IRRs: five (2.40%) with grade 1, twenty-eight (13.46%) with grade 2, and eight (3.85%) with grade 3. The 41 patients had 71 IRRs, mainly fever (40.85%), chest pain/tightness (12.68%), and dyspnea (9.86%). The occurrence of IRRs in the first infusion was significantly lower in patients who received oral acetaminophen prophylaxis than those who did not (10.72% *vs* 30.21%, P<0.001). For the subsequent cycles with 90-min SDI, only two (0.25%) IRRs occurred among 814 infusions (one grade 1 hand numbness and one grade 2 chill/fever). The 90-min obinutuzumab SDI might be safe and feasible in patients with B-cell NHL in China.

## Introduction

Lymphoma is one of the most common hematological malignancies in China. According to the World Health Organization GLOBOCAN 2020, there were 6829 new cases of Hodgkin lymphoma (HL) in China in 2020 and 2807 related deaths, and there were 92,834 new cases of non-Hodgkin lymphoma (NHL) and 54,351 related deaths (1). With the development of standard chemotherapy and immunotherapy, the survival of lymphoma patients has greatly improved. The 5-year overall survival (OS) of patients with lymphoma in some large medical centers in China has reached 65% (2). Among mature B-cell lymphoma, the 5-year OS was highest in follicular lymphoma (77.8%), followed by Burkitt lymphoma (76.5%), marginal zone lymphoma (74.1%), diffuse large B-cell lymphoma (61.5%), small lymphocytic lymphoma/chronic lymphocytic leukemia (55.1%), and mantle cell lymphoma (44.3%), close to the level of developed countries in Europe and the United States of America (USA) (2).

Obinutuzumab is a novel humanized type II anti-CD20 monoclonal antibody approved in the USA and China for treating B-cell malignancies (3,4). *In vitro*, the obinutuzumab-induced antibody-dependent cell-mediated cytotoxicity is 35-100 times higher than rituximab, and obinutuzumab induces potent complement-dependent cytotoxicity and cell death (5,6). After 8 years of follow-up, the GALLIUM study demonstrated that obinutuzumab was associated with significantly higher progression-free survival (PFS) than with rituximab in previously untreated patients with follicular lymphoma (FL) (7). Large-scale clinical studies of obinutuzumab yielded promising efficacy and safety in treating CD20-positive B-cell NHL (8-10). In Europe and the USA, obinutuzumab has been approved for the treatment of FL and chronic lymphocytic leukemia (CLL); obinutuzumab is also recommended by the National Comprehensive Cancer Network (NCCN) guidelines for the treatment of a variety of CD20-positive B-cell NHL (11,12).

The standard intravenous administration protocol of obinutuzumab typically takes more than 3-4 h (3). Prolonged intravenous infusions are burdensome and inconvenient for the patients, require a longer observation time and increase the workload of healthcare professionals. The NCCN, Chinese Society of Hematology, and Chinese Medical Association issued guidelines for the rapid infusion of rituximab (12,13). They recommend that patients without infusion-related reactions (IRRs) after the first rituximab infusion can receive subsequent infusions over 90-min. A shorter duration of infusion (SDI) can reduce treatment burden and improve healthcare resource utilization, and the principles applied to rituximab might also be applicable to obinutuzumab. Previous studies outside China demonstrated that obinutuzumab SDI in cycle 2 or more improved patient convenience and treatment room efficiency, with good safety and effectiveness (14-16). Despite the approval of obinutuzumab in more than 100 countries/regions worldwide, it was only recently approved in China, and the experience with its use is still limited. In addition, there are no reports of obinutuzumab SDI despite a high patient demand in China.

Therefore, this study aimed to examine the safety and feasibility of 90-min obinutuzumab SDI in patients with B-cell NHL in a tertiary hospital in China, as well as two infusion protocols with different drug dilutions and IRR prophylaxis. The results could help determine the proper administration of obinutuzumab.

## Material and Methods

### Study design and patients

This prospective, single-center, open-label clinical trial was performed with patients with B-cell NHL at Jiangsu Province Hospital (China). In the 2022 Annual Science and Technology Evaluation Index of China's hospitals, the Hematology Department of Jiangsu Province Hospital ranks 9th in 1644 tertiary hospitals, which has a certain influence in China. The trial was approved by the Ethics Committee of Jiangsu Province Hospital (#2022-SR-169) and was conducted in accordance with the Declaration of Helsinki and Good Clinical Practice guidelines. All participants signed the study informed consent form. This trial was registered at ClinicalTrials.gov (#NCT05510219).

Patients with B-cell NHL who visited the Department of Hematology of Jiangsu Province Hospital between November 2021 and December 2022 were screened. The inclusion criteria were: 1) diagnosis of B-cell lymphoma in accordance with the World Health Organization guidelines for the diagnosis of lymphoma (2016 version) (17); 2) indications for obinutuzumab treatment; 3) no significant organ damage, 4) Eastern Cooperative Oncology Group (ECOG) performance status 0-2 and; 5) life expectancy ≥6 months. The exclusion criteria were: 1) pregnant or lactating; 2) positive for human immunodeficiency virus or hepatitis B virus infection and had not received anti-viral treatment; 3) severe hepatic or renal insufficiency, or 4) severe cardiovascular disease.

### Obinutuzumab intravenous infusion protocol

According to the Chinese Expert Consensus on Clinical Use Guidelines for Obinutuzumab (2021 Edition) and instruction manual of obinutuzumab, all patients received six or eight cycles of a combination of obinutuzumab (1000 mg on day 1 and additional doses on days 8 and 15 of cycle 1) and chemotherapy in accordance. According to the different chemotherapy combinations, a cycle lasted 21 or 28 days. The patients who achieved complete or partial remission after 6-8 cycles continued to receive obinutuzumab (1000 mg) monotherapy as a maintenance treatment every 2 months until disease progression or for up to 2 years. All patients were treated with the standard intravenous infusion regimen for the first infusion (cycle 1, day 1). For the patients without grade ≥3 IRRs during the first infusion, a 90-min SDI was used for the subsequent infusions (i.e., cycle 1, days 8 and 15, and for cycle 2 and subsequent infusions).

The standard infusion was initiated at a rate of 50 mg/h, and the infusion rate was increased by 50 mg/h every 30 min until a maximum rate of 400 mg/h was reached. For a subsequent infusion, if the final rate of the previous infusion was ≥100 mg/h without IRR or with grade 1 IRR, the infusion was initiated at 100 mg/h and increased by 100 mg/h every 30 min until a maximum rate of 400 mg/h was reached. If a grade ≥2 IRR occurred during the previous infusions, the new infusion was initiated at 50 mg/h and increased by 50 mg/h every 30 min until a maximum rate of 400 mg/h was reached.

In 90-min SDI, 20% of the obinutuzumab solution (50 mL) was infused over the first 30 min, followed by the remaining 80% of the obinutuzumab solution (200 mL) infused over the next 60 min, for a total infusion duration of 90 min, according to the Chinese expert consensus on rapid infusion of rituximab (13).

### Drug dilution and IRR prophylaxis

The patients were grouped into two cohorts based on patient wishes and the investigator's discretion. The study personnel performing the assessments were not involved in implementing any aspect of the intervention and knew the participants only by their study identification number.

The two cohorts had the same infusion protocol and only differed in the dilution of obinutuzumab for the standard infusion and IRR prophylaxis. In cohort 1, 100 mg of obinutuzumab was diluted in 250 mL of saline (0.4 mg/mL), and 900 mg of obinutuzumab was diluted in 250 mL of saline (3.6 mg/mL) for the standard infusion. In cohort 2, 100 mg of obinutuzumab was diluted in 100 mL of saline (1 mg/mL), and 900 mg of obinutuzumab was diluted in 500 mL of saline (1.8 mg/mL). In cohort 1, the IRR prophylaxis for all infusions included intravenous dexamethasone (5 mg), intramuscular promethazine (25 mg), and oral acetaminophen. In cohort 2, intravenous dexamethasone (5 mg) and intramuscular promethazine (25 mg) were given as IRR prophylaxis in the first infusion; the subsequent infusion was given with intravenous dexamethasone (5 mg) only (Supplementary Table S1).

### Endpoints

The primary endpoint was the incidence of IRR during the standard infusion and 90-min SDI regimens. The severity of an IRR was graded according to the National Cancer Institute Common Terminology Criteria for Adverse Events version 5.0 (18). Grade 1: the reaction is transient and mild, requiring no infusion interruption and no treatment. Grade 2: infusion needs to be interrupted, and symptomatic treatment should be given promptly (e.g. antihistamines or non-steroidal anti-inflammatory drugs), requiring prophylactic medication for no longer than 24 h. Grade 3: the management of the condition is delayed (e.g. with no immediate symptomatic treatment and/or brief interruption of infusion), and the patient develops relapse after initial symptom improvement, with clinical sequelae requiring hospitalization. Grade 4: the reaction is life-threatening, requiring urgent treatment. Grade 5: death. IRRs are the most common adverse events associated with the combination of obinutuzumab and chemotherapy, often occurring within 24 h of infusion.

The secondary endpoints were the IRR types and duration of obinutuzumab administration by cycle. IRR symptoms mainly include nausea, vomiting, diarrhea, fever, chills, respiratory symptoms (e.g., bronchospasm, throat irritation, wheezing, and laryngeal edema), and cardiac symptoms (e.g., atrial fibrillation).

### Statistical analysis

This was an exploratory clinical trial with no hypothesis testing, and the study was performed without power analysis. Excel (Microsoft, USA) and SPSS 23.0 (IBM, USA) were used for data entry and analysis. The average incidence of IRR during each treatment cycle of obinutuzumab infusion is reported as n (%) and was analyzed using the chi-squared test. Age is reported as means±SD and was analyzed using Student's *t*-test. Two-sided P-values <0.05 were considered statistically significant.

## Results

### Characteristics of the patients

This study enrolled 208 patients, 112 in cohort 1 and 96 in cohort 2. The patients were 54.38±12.45 years of age. There were 117 (56.25%) males and 91 (43.75%) females. The patients had FL (49.52%), CLL (16.83%), diffuse large B-cell lymphoma (DLBCL) (16.83%), or other types of NHL (16.83%). Most patients had stage IV disease (64.42%) (Table 1). There were no significant differences between the two cohorts except that cohort 2 had a higher proportion of patients with FL (61.46 *vs* 39.29%, P=0.012).

**Table 1:** Baseline clinical characteristics.

Characteristics	All (n=208)	Cohort 1 (n=112)	Cohort 2 (n=96)	P
Age (years)	54.38±12.45	53.71±12.57	55.16±12.33	0.781
Gender, n (%)				0.142
Male	117 (56.25)	66 (58.93)	51 (53.13)	
Female	91 (43.75)	46 (41.07)	45 (46.88)	
Pathological classification, n (%)				0.012
Follicular lymphoma	103 (49.52)	44 (39.29)	59 (61.46)	
Chronic lymphocytic leukemia	35 (16.83)	33 (29.46)	2 (2.08)	
Diffuse large B-cell lymphoma	35 (16.83)	15 (13.39)	20 (20.83)	
Others	35 (16.83)	20 (17.86)	15 (15.63)	
Disease stage, n (%)				0.123
I	8 (0.38)	6 (5.36)	2 (2.08)	
II	24 (11.54)	13 (11.61)	11 (11.46)	
III	41 (19.71)	24 (21.43)	17 (17.71)	
IV	134 (64.42)	69 (61.61)	65 (67.71)	
V	1 (0.48)	0	1 (1.04)	
ECOG PS, n (%)				0.114
0	93 (44.71)	35 (31.25)	58 (60.42)	
1	104 (50.00)	70 (62.50)	34 (35.42)	
2	11 (5.29)	7 (6.25)	4 (4.17)	

ECOG PS: Eastern Cooperative Oncology Group performance status. Data are reported as n (%) except for age (mean and SD). Chi-squared test and Student’s *t*-test.

### Occurrence of IRRs in the first infusion

All 208 patients completed the first infusion of obinutuzumab, among which 41 patients (19.71%) had IRRs: five (2.40%) with grade 1, twenty-eight (13.46%) with grade 2, and eight (3.85%) with grade 3. The 41 patients had 71 IRRs, mainly fever (40.85%), chest pain/tightness (12.68%), and dyspnea (9.86%) (Table 2).

**Table 2:** Occurrence of IRRs in cycle 1 and in subsequent cycles.

	Cycle 1, day 1	Second and subsequent cycles	
	Standard infusion	Standard infusion	90-min SDI
Total number of infusions	208	6	814
IRR incidence, n (%)	41 (19.71)	0	2 (0.25)
Grade 1	5 (2.40)	0	1 (0.12)
Grade 2	28 (13.46)	0	1 (0.12)
Grade 3	8 (3.85)	0	0
Grade 4	0	0	0
Grade 5	0	0	0
Manifestations, n (%)	71	0	2
Chill/fever	29 (40.85)	0	1 (50.00)
Hand numbness	0	0	1 (50.00)
Rash/pruritus	3 (4.23)	0	0
Headache/dizziness	1 (1.41)	0	0
Chest pain/chest tightness	9 (12.68)	0	0
Dyspnea	7 (9.86)	0	0
Laryngeal edema	3 (4.23)	0	0
Nausea/vomiting	4 (5.63)	0	0
Abdominal pain/diarrhea	4 (5.63)	0	0
Blood pressure drop	5 (7.04)	0	0
Heart rate changes	2 (2.82)	0	0
Urinary incontinence	1 (1.41)	0	0
Unconsciousness	1 (1.41)	0	0
Low back pain	2 (2.82)	0	0

SDI: shorter duration of infusion; IRR: infusion-related reactions. Data are reported as n (%).

In cohort 1, 12 (10.72%) IRRs occurred: four (3.58%) with grade 1, six (5.36%) with grade 2, and two (1.79%) with grade 3. In cohort 2, twenty-nine (30.21%) IRRs occurred: one (1.04%) grade 1, twenty-two (22.92%) grade 2, and six (6.25%) grade 3. The occurrence of IRRs was significantly higher in cohort 2 than in cohort 1 during the first infusion of obinutuzumab (30.2 *vs* 10.72%, P<0.001) (Table 3).

**Table 3:** Summary of IRRs and types between the two cohorts.

	Cycle 1, day 1			Second and all other cycles					
	Standard infusion			Standard infusion			90-min SDI		
	Cohort 1	Cohort 2	P	Cohort 1	Cohort 2	P	Cohort 1	Cohort 2	P
Total number of infusions	112	96		1	5		422	392	
IRR incidence, n (%)	12 (10.72)	29 (30.21)	<0.001	0	0	>0.999	1 (0.24)	1 (0.26)	>0.999
Grade 1	4 (3.58)	1 (1.04)		0	0		0	1 (0.26)	
Grade 2	6 (5.36)	22 (22.92)		0	0		1 (0.24)	0	
Grade 3	2 (1.79)	6 (6.25)		0	0		0	0	
Grade 4	0	0		0	0		0	0	
Grade 5	0	0		0	0		0	0	
Manifestations, n (%)	15	56		0	0		1	1	
Chill/fever	9 (60.00)	20 (35.71)		0	0		1 (100)	0	
Hand numbness	0	0		0	0		0	1 (100)	
Rash/Pruritus	1 (6.67)	2 (3.57)		0	0		0	0	
Headache/dizziness	0	1 (1.79)		0	0		0	0	
Chest pain/chest tightness	2 (13.33)	7 (12.5)		0	0		0	0	
Dyspnea	0	7 (12.5)		0	0		0	0	
Laryngeal edema	0	3 (5.36)		0	0		0	0	
Nausea/vomiting	1 (6.67)	3 (5.36)		0	0		0	0	
Abdominal pain/diarrhea	1 (6.67)	3 (5.36)		0	0		0	0	
Blood pressure drop	1(6.67)	4 (7.14)		0	0		0	0	
Heart rate changes	0	2 (3.57)		0	0		0	0	
Urinary incontinence	0	1 (1.79)		0	0		0	0	
Unconsciousness	0	1 (1.79)		0	0		0	0	
Low back pain	0	2 (3.57)		0	0		0	0	

SDI: shorter duration of infusion; IRR: infusion-related reactions. Data are reported as n (%). Chi-squared test.

### Occurrence of IRRs in the second and subsequent infusions

**Figure 1:**
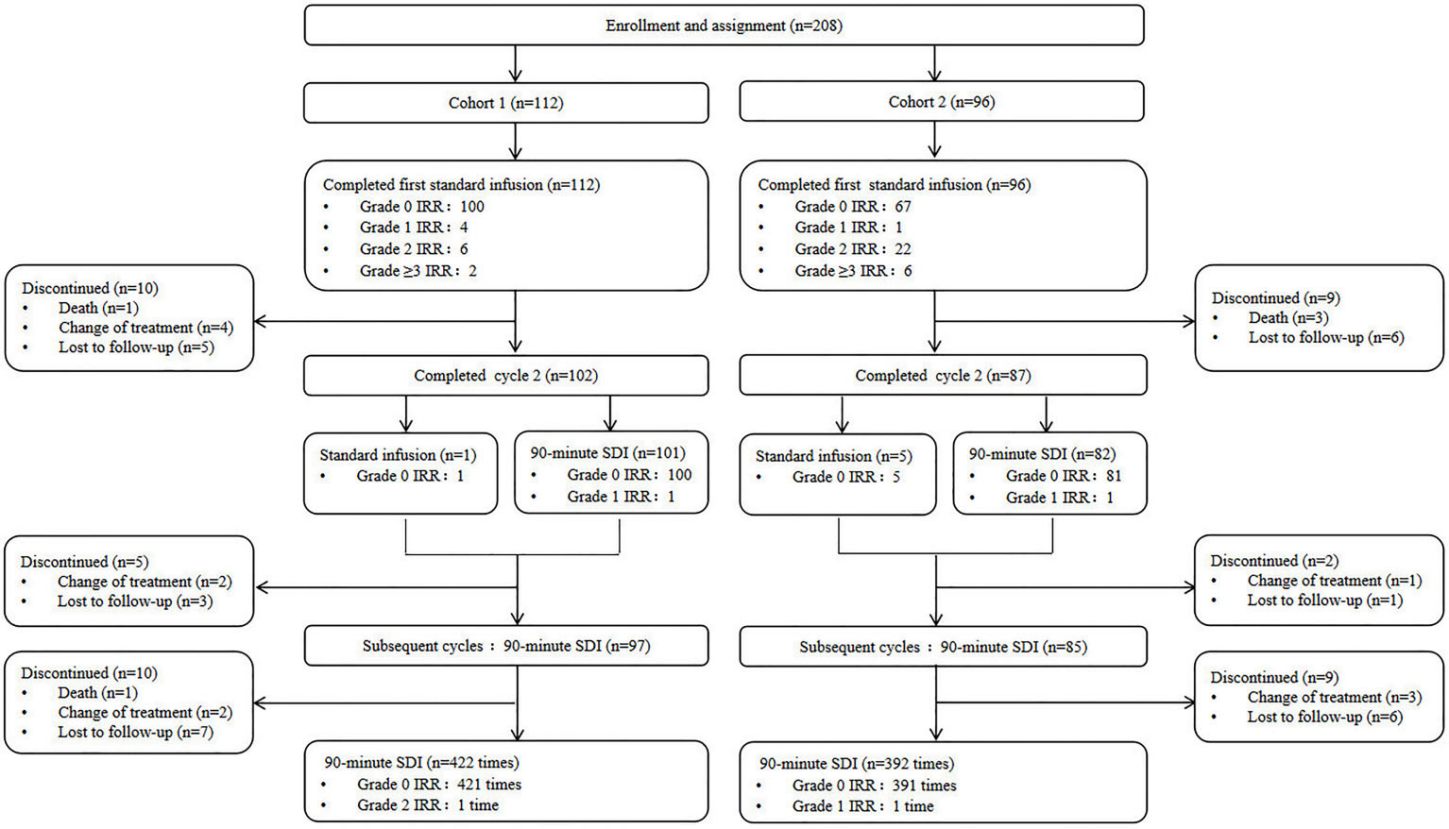
Patient flow. SDI: shorter duration of infusion; IRR: infusion-related reactions.

In cohort 1, one patient died, four changed treatment, and five were lost to follow-up after the first infusion. In cohort 2, three patients died, and six were lost to follow-up (Figure 1). Six patients (1 in cohort 1 and 5 in cohort 2) still received the standard infusion in the second infusion of obinutuzumab, and no IRRs occurred.

For the second and subsequent cycles with 90-min SDI, only two (0.25%) IRRs occurred among 814 infusions: one grade 1 hand numbness in cohort 2 and one grade 2 chill/fever in cohort 1 (Tables 2 and 3).

## Discussion

This study aimed to analyze the safety of 90-min obinutuzumab SDI in patients with B-cell NHL. In patients without IRR grade ≥3 in the first standard infusion of obinutuzumab, the 90-min SDI was given 814 times and only two (0.25%) IRRs occurred. The occurrence of IRRs in the first infusion was significantly higher in cohort 2 than in cohort 1, which differed in the dilution and additional oral acetaminophen prophylaxis. This study is the first reported China-based study on the safety of obinutuzumab SDI in treating patients with B-cell NHL, which included a variety of diseases, including FL, CLL, DLBCL, and other types.

IRRs are troublesome events that can decrease the patient's quality of life, decrease their compliance, lead to treatment discontinuation, and affect the disease prognosis. Some IRRs can also be life-threatening (19). IRR is the most common adverse event associated with administering a combination of obinutuzumab and chemotherapy, and the incidence of IRRs may be related to cytokine release due to B-cell clearance (20,21). The high affinity of obinutuzumab to the FcγIII receptors on effector cells due to the structural modification of obinutuzumab by glycosylation engineering leads to an increased potential for cytokine release, which might contribute to the high rates of IRRs during obinutuzumab infusion, especially the first infusion (20,22-25).

Still, a major issue with obinutuzumab treatment is the long infusions (3-4 h) that limit the activity of patients, causes fatigue, anxiety, irritability, and other discomfort symptoms, and increases the workload of the medical staff. Hence, for patients who do not experience significant adverse events during the first infusion, some studies of rapid obinutuzumab infusion have been conducted in non-Chinese areas to improve patient convenience and save healthcare resources. The GAZELLE international, open-label, phase IV clinical trial conducted by Canales et al. (14) evaluated the safety and efficacy of a 90-min obinutuzumab SDI in previously untreated patients with FL. They reported no new safety signals with obinutuzumab SDI, the remission data at the end of induction therapy were consistent with those of previous studies, no IRRs grade ≥3 were observed in the second cycle, and only one grade 3 IRR was observed with obinutuzumab SDI in the subsequent cycles. In another phase II multicenter, open-label clinical trial, patients with CD20-positive B-cell NHL were treated with a rapid infusion of obinutuzumab that showed similar tolerability and pharmacokinetic profile to the conventional administration (15). In addition, the GATHER study, a phase II, open-label, multicenter, single-arm clinical study, showed that a 120 or 90-min rapid infusion of obinutuzumab showed good tolerability and no grade 3 IRRs in previously untreated patients with CD20-positive advanced DLBCL (16). Furthermore, in the present study, a 90-min obinutuzumab SDI showed similar results without new safety signals.

Despite the differences in obinutuzumab dilution and IRR prophylaxis before infusion between the two cohorts, the patients in both cohorts 1 and 2 experienced IRRs in the first treatment cycle, and none experienced IRRs grade ≥3 with obinutuzumab SDI in the subsequent cycles. In the first cycle, the occurrence of IRRs was significantly higher in cohort 2 than in cohort 1. Besides different dilutions, the patients in cohort 2 did not receive acetaminophen before infusion. Whether omitting acetaminophen resulted in the higher occurrence of IRRs remains to be determined. Cohort 2 also included more patients with FL than cohort 1. Since the patients in the two cohorts were not randomly assigned, and the baseline was not comparable, both cohorts appeared to be safe and feasible, but comparisons should be taken with caution until confirmed.

This study had limitations. The number of patients in this study was small, and the observation period was short because obinutuzumab has been marketed in China for a relatively short time. Thus, the current results need to be further validated. It is necessary to expand the study population in the future to assess prospectively and dynamically the safety and feasibility of obinutuzumab SDI. In addition, due to time constraints, this study only focused on IRRs that occurred during the infusion of obinutuzumab as an induction treatment of B-cell NHL, while the long-term safety and therapeutic efficacy need to be examined through further follow-up and observation. Finally, the SDI decreased the infusion time of obinutuzumab, but the patient's quality of life and preferences were not evaluated.

### Conclusion

This study is the first to report the safety and feasibility of a 90-min obinutuzumab SDI in patients with B-cell NHL in a tertiary hospital in China. Future studies are required to expand the sample size and follow-up time to provide stronger evidence to support the subsequent use of the rapid infusion regimen of obinutuzumab as a routine immunochemotherapy option for B-cell lymphoma in China.

## Supplementary Material

Click here to view [pdf].
