# Supplementary material for: Safety of a 90-min duration of intravenous infusion of obinutuzumab in patients with B-cell non-Hodgkin's lymphoma in a tertiary hospital in China: a prospective, open-label, exploratory clinical trial
Source: Braz J Med Biol Res. 2024 Feb 9;57:e13284. doi: 10.1590/1414-431X2023e13284 (PMC10868189; doi:10.1590/1414-431X2023e13284)
Supplement: Supplementary file 1 [file 1414-431X-bjmbr-57-e13284-suppl1.pdf]

Table S1. Obinutuzumab dilution and infusion-related reactions (IRR) prophylaxis.

	First infusion (cycle 1, day 1)		Second and all other infusions	
	Drug dilution	IRR prophylaxis	Drug dilution	IRR prophylaxis
Cohort 1	100 mg of obinutuzumab in 250 mL of 0.9% normal saline (0.4 mg/mL solution), and 900 mg of obinutuzumab in 250 mL of 0.9% normal saline (3.6 mg/mL solution) on the following days	Intravenous dexamethasone 5 mg, intramuscular promethazine 25 mg, and oral acetaminophen 30–60 min before each obinutuzumab infusion	1000 mg of obinutuzumab in 250 mL of 0.9% saline (4 mg/mL solution)	Intravenous dexamethasone 5 mg, intramuscular promethazine 25 mg, and oral acetaminophen 30–60 min before each obinutuzumab infusion
Cohort 2	100 mg of obinutuzumab in 100 mL of 0.9% normal saline (1 mg/mL solution), and 900 mg of obinutuzumab in 500 mL of 0.9% normal saline (1.8 mg/mL solution)	Intravenous dexamethasone 5 mg and intramuscular promethazine 25 mg 30-60 min before each obinutuzumab infusion	1000 mg of obinutuzumab in 250 mL of 0.9% saline (4 mg/mL solution)	Intravenous dexamethasone 5 mg 30–60 min before each obinutuzumab infusion

SDI: shorter duration of infusion.
